# M6A-related bioinformatics analysis reveals that HNRNPC facilitates progression of OSCC via EMT

**DOI:** 10.18632/aging.103333

**Published:** 2020-06-11

**Authors:** Guang-Zhao Huang, Qing-Qing Wu, Ze-Nan Zheng, Ting-Ru Shao, Yue-Chuan Chen, Wei-Sen Zeng, Xiao-Zhi Lv

**Affiliations:** 1 Department of Oral and Maxillofacial Surgery, NanFang Hospital, Southern Medical University, Guangzhou, China; 2 Department of Cell Biology, School of Basic Medical Science, Southern Medical University, Guangzhou, China

**Keywords:** oral squamous cell carcinoma, m6A, HNRNPC, prognosis, EMT

## Abstract

Increasing evidence suggests that N6-methyladenosine(m6A) has a vital role in cancer progression. Therefore, we aimed to explore the prognostic relevance of m6A-related genes in oral squamous cell carcinoma (OSCC). First, Expression profiles were downloaded from The Cancer Genome Atlas (TCGA) and m6A-related genes were extracted afterwards. Then, cluster analysis and principal component analysis (PCA) were used to analyze m6A-related genes. And differentially-expressed analysis was performed in R software. Furthermore, a risk model was constructed, and crucial m6A genes were selected to explore its biological effects in OSCC cells. Total of 13 m6A-related genes were extracted and 8 differentially-expressed genes were identified. Subsequently, m6A-based clustering showed 2 subtypes with different clinical outcome. In addition, a risk model was successfully established. Of 13 m6A-related genes, only heterogeneous nuclear ribonucleoprotein C (HNRNPC) might be an independent biomarker and mean unfavorable overall survival in OSCC by univariate and multivariate cox regression analysis. Functional studies revealed that overexpression of HNRNPC promoted carcinogenesis of OSCC via epithelial- mesenchymal transition (EMT). In total, a risk model of m6A-related genes in OSCC was established. Subsequently, HNRNPC was proved to promote OSCC carcinogenesis and be an independent biomarker prognostic biomarker of OSCC, suggesting that it might be a new biomarker and therapeutic target of OSCC.

## INTRODUCTION

As the most common oral cancer, oral squamous cell carcinoma (OSCC) is a serious global problem because of its most severe impact on life quality of patients [1]. Clinical data indicate that smoking, drinking and betel nut consumption are the main causes of the high incidence of OSCC. Recently, Leemans et al. demonstrated that human papillomavirus (HPV) is also considered as one of the potential risk factors of OSCC [2]. In spite of advancement in diagnosis and therapeutic methods, the prognosis of OSCC has not improved obviously over the past few years. High recurrence rate and lymph node metastasis risk lead to an unsatisfactory 5-year overall survival rate, which ranges from 45 to 50% [3]. Therefore, it is imperative to further understand the potential mechanism of the initiation and progression in OSCC.

More than 160 different chemical modifications in RNA have been identified in all living organisms [4]. Among these RNA modifications, N6-methyladenosine (m6A), methylated at the N6 position of adenosine, is the most widespread internal modification. M6A is methylated by methyltransferase, removed by demethylase and recognized by m6A binding protein and they are jargonized as ‘writers’, ‘erasers’ and ‘readers’ [5]. Researches demonstrated that m6A modification was significantly associated with the abnormal expression proto-oncogenes and tumor suppressor genes [6, 7]. It has opened a new chapter in cancer research to explore the role of m6A. M6A plays a significant role in translation [8], splicing [9], stability [10], degradation, transcript, nuclear transport [11]. Recent studies indicate that m6A modification is related to tumorigenesis [12], proliferation [13], invasion [14] and metastasis [15]. In OSCC, research has indicated that METTL3 enhanced OSCC tumorigenesis through YTHDF1-mediated m6A modification [16]. However, there are fewer studies to explore the m6A prognostic value in OSCC through analyzing m6A-related genes.

In this study, we aim to analyze the differentially-expressed profiles of m6A-related genes in OSCC and establish a cox regression model to predict the overall survival. In addition, total m6A levels in total RNAs were detected in OSCC and normal adjacent tissues. Finally, HNRNPC was identified as an independent prognostic biomarker in OSCC and its tumorigenic roles was explored in vitro. This study may provide a new therapeutic target for OSCC.

## RESULTS

### Identification of m6A-related genes differentially expressed analysis

**Figure 1:**
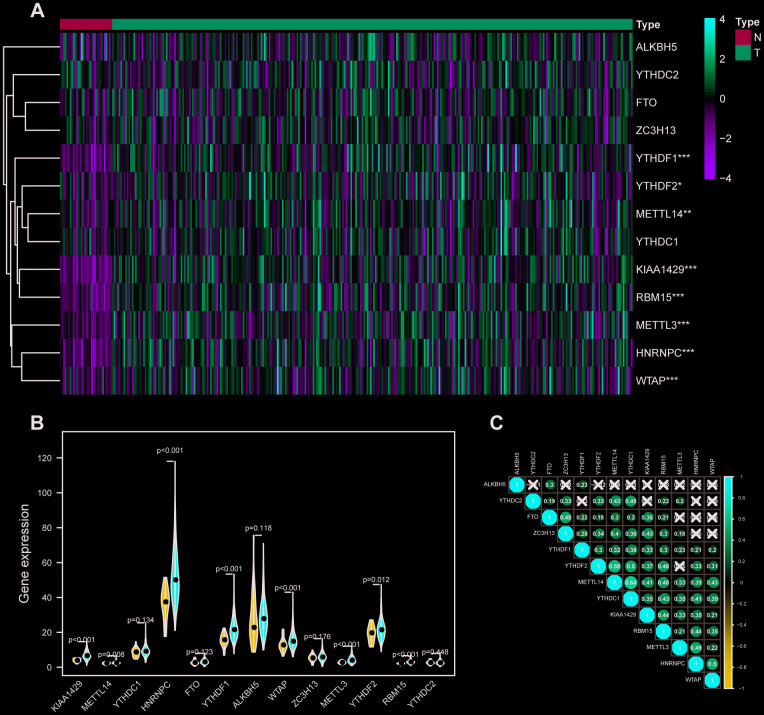
**M6A-related genes expression level and correlation in OSCC.** (**A**) 317 OSCC samples and 32 normal control m6A expression level on basis of TCGA database. N stands for normal control, while T represents tumor samples. Differences were considered significant at p <0.05 *; p <0.01**; p <0.001***.The ascending normalized expression level in the heatmaps is colored from green to red. (**B**) Differently expressed analysis of 13 m6A related genes. Blue stands for normal control, while red OSCC samples. (**C**) The correlation between 13 m6A related genes. The ascending normalized correlation level in the picture is colored from blue to red.

RNA expression profiles and their corresponding clinical data of 317 OSCC samples and 32 normal samples were downloaded from TCGA database, and the raw data were normalized in a log2(x + 1) manner. Then, expression profiles of 13 m6A-related genes including METTL3, METTL14, WTAP, KIAA1429, RBM15, ZC3H13, YTHDC1, YTHDC2, YTHDF1, YTHDF2, HNRNPC, FTO, ALKBH5 were extracted from the transcriptome data and 8 differentially expressed m6A-related genes were identified (Figure 1A, 1B). Furthermore, the correlation between m6A related genes was performed in R software (Figure 1C). HNRNPC was correlated with 7 of 13 m6A-related genes including METTL3, METTL14, WTAP, KIAA1429, RBM15, YTHDC1, YTHDF1, YTHDF2. Interestingly, METTL14 had the strongest correlation with YTHDC1.

### M6A-based clustering showed 2 subtypes of OSCC

**Figure 2:**
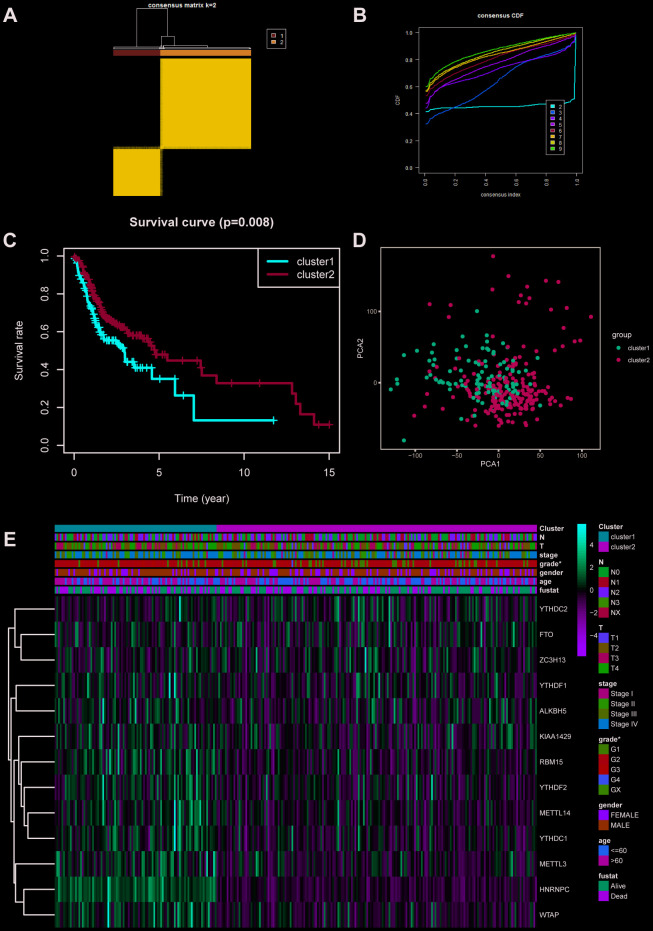
**Cluster analysis based on m6A-related genes.** (**A**, **B**) Cluster analysis indicated that 317 OSCC samples in TCGA can divided into 2 groups. (**C**) Survival analysis between cluster1 and 2. (**D**) Principal component analysis was performed on basis of cluster analysis. PCA1 represents principal component analysis 1, while PCA2 stands for principal component analysis 2. (**E**) The correlation of cluster analysis and clinical characteristics (grade, p=0.0352). N stands for N classification in TNM system, and T stands for T classification in TNM system.

According to the expression profiles of m6A-related genes, cluster analysis was performed to analyze the 317 OSCC samples from the TCGA database, and 2 subtypes were determined (Figure 2A–2C). Subsequently, the result of principal component analysis (PCA) indicates that m6A-related genes can distinguish OSCC patients (Figure 2D). On basis of correlation analysis of clinical characteristics (Figure 2E), the differentiation grade of subtype1 is lower than subtype2 and indicates unfavorable overall survival rate. Unfortunately, there are no correlation between the cluster analysis and other clinical parameters.

### Establishment of cox regression model

**Figure 3:**
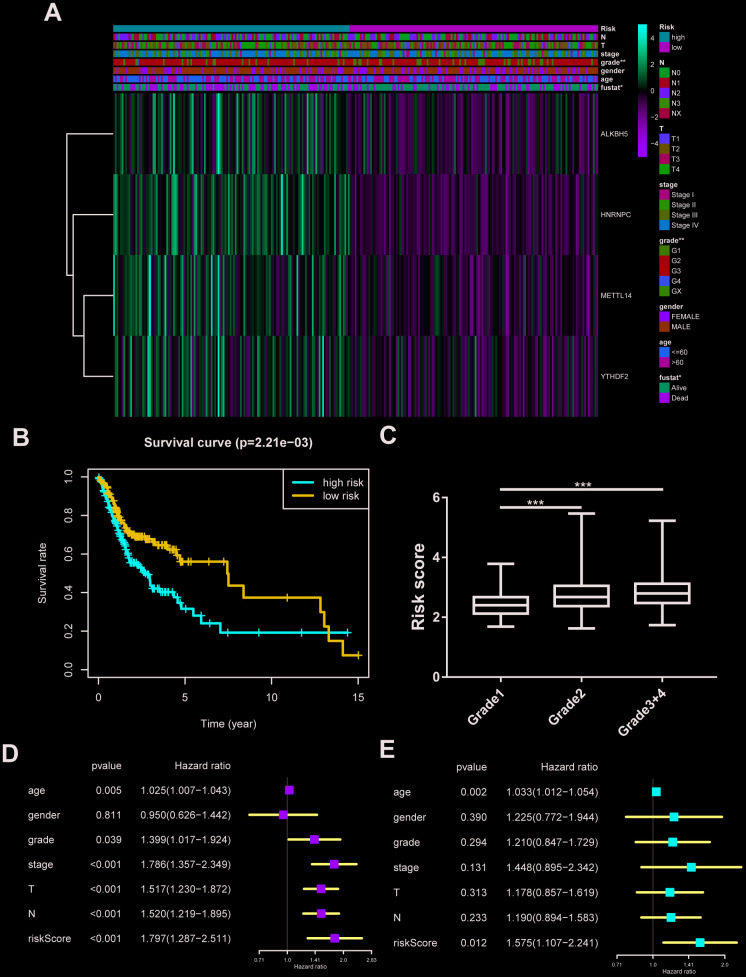
**Construction of cox regression model.** (**A**) The heatmap performed in R software on basis of risk score level and clinical characteristics (grade p=0.003). N stands for N classification in TNM system, and T stands for T classification in TNM system. (**B**) Survival analysis based on risk score. (**C**) The risk score distribution of differentiation grades. The y axis represent risk score level and x axis represent different grade (grade1 vs grade2, p=0.0002; grade1 vs grade3+4, p=0.0001). (**D**, **E**) Univariate cox regression and multivariate cox regression according to risk score and clinical characteristics. N stands for N classification in TNM system, and T stands for T classification in TNM system.

All m6A related genes were enrolled in univariate and multivariate cox regression, and HNRNPC might be an independent biomarker in OSCC (Supplementary Figure 1A, 1B). Furthermore, LASSO cox regression along with 10-fold cross-validation was performed to determine variables. Finally, 4 variables including HNRNPC, METTL14, YTHDF2, ALKBH5 were selected in cox regression (Supplementary Figure 1C, 1D). Risk score =(0.00815*HNRNPC)+(0.0482*METTL14)+(0.0081* YTHDF2)+(0.0086*ALKBH5). And risk score was visualized in R software (Supplementary Figure 1E, 1F). Subsequently, the OSCC patients were divided into high risk and low risk group on basis of median value of risk model (Figure 3A). Survival package in R software was used to analyze the 2 groups. Low risk group tended to experience longer survival time (Figure 3B), and risky genes were higher in high risk group. Correlation analysis between the clinical traits and risk level indicated that high risk group meant lower differentiation grade (Figure 3C). And independent prognostic analysis indicated that risk score may be an independent prognostic biomarker (Figure 3D, 3E).

### Abnormal m6A quantification level and HNRNPC expression in OSCC

**Figure 4:**
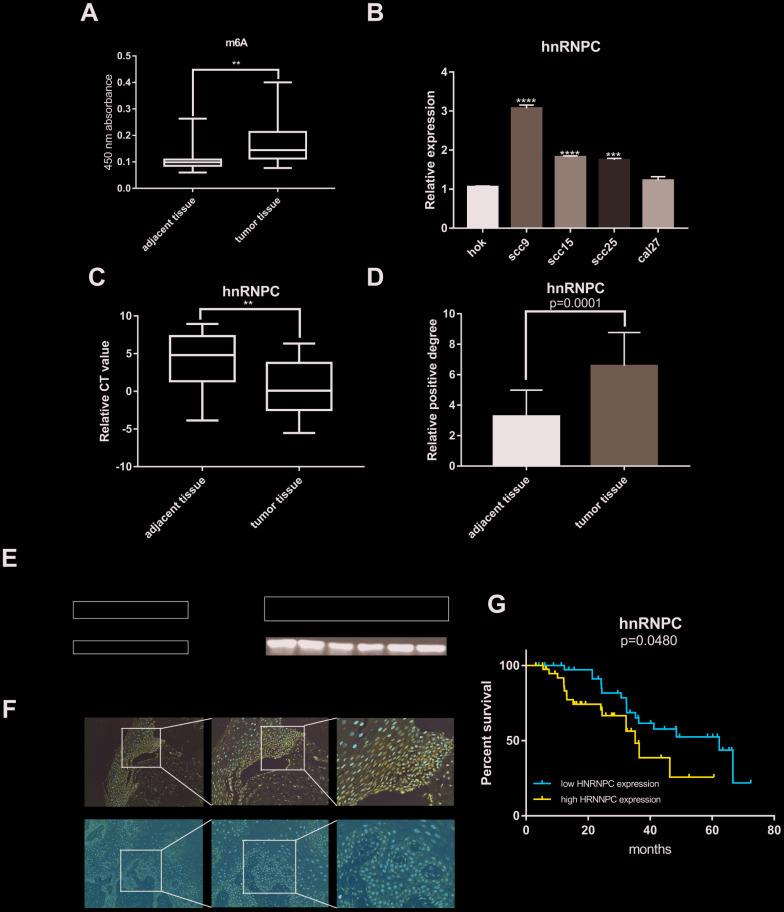
**M6A and HNRNPC expression level in OSCC.** (**A**) M6A level was detected in 80 pairs OSCC tissues and adjacent normal tissues (p=0.0047). (**B**, **C**) MRNA level of HNRNPC was detected in 4 OSCC cell lines (scc9 p<0.0001, scc15 p<0.0001, scc25 p=0.0002) and 80 pairs OSCC tissues (p=0.0038). (**D**) Statistical analysis of immunohistochemistry in OSCC tissues (p=0.0001). (**E**) HNRNPC protein level in OSCC cell lines and tissues. (**F**) The represent results of immunohistochemistry. (**G**) Survival analysis of HNRNPC was performed in 80 OSCC samples from Nanfang Hospital (p=0.048).

Plenty of evidence has shown that m6A RNA methylation promote tumor initiation and progression. However, there are fewer studies to explore the role of m6A-related genes in OSCC. Therefore, m6A RNA methylation quantification kit was used to detect the m6A level in OSCC and normal adjacent tissues. The result showed that m6A level upregulated in tumor tissues compared with normal adjacent tissues (Figure 4A). Furthermore, univariate and multivariate cox regression analysis indicated that HNRNPC may be an independent biomarker in OSCC (Supplementary Figure 1A, 1B). Besides, HNRNPC was significantly relevant to overall survival rate in TCGA (Supplementary Figure 2A). Consequently, HNRNPC protein level and mRNA level were detected in OSCC tissues and cell lines (Figure 4B, 4C, 4E). Immunohistochemistry assay also indicated that HNRNPC was upregulation in OSCC (Figure 4D, 4F). Furthermore, the relationship between HNRNPC expression levels and clinical parameters in individuals with OSCC (Table 1) showed that higher HNRNPC expression levels were positively correlated with advanced clinical stage (p=0.0448) and lymph node metastasis (p=0.0431). Moreover, high HNRNPC mRNA level meant undesirable overall survival in 80 OSCC samples (Figure 4G).

**Table 1:** Correlation between HNRNPC expression and clinical parameters in OSCC patients (n=80).

**Variables**	**n**	**HNRNPC(%)**		**p value**
		**High expression**	**Low expression**	
Age(years)				0.6191
>=60	22	12	10	
<60	58	27	31	
Gender				0.5828
male	61	41	20	
female	19	11	8	
Stage				**0.0448**
I + II	46	19	27	
III + IV	34	22	12	
T classification				0.4014
1	10	4	6	
2	52	30	22	
3	6	4	2	
4	12	9	3	
N classification				**0.0431**
N0+N1	59	27	32	
N2+N3	21	15	6	
Distant metastasis				0.1372
M0	62	29	33	
M1	18	12	6	

### HNRNPC promotes OSCC proliferation, migration, invasion and EMT (epithelial-mesenchymal transition)

**Figure 5:**
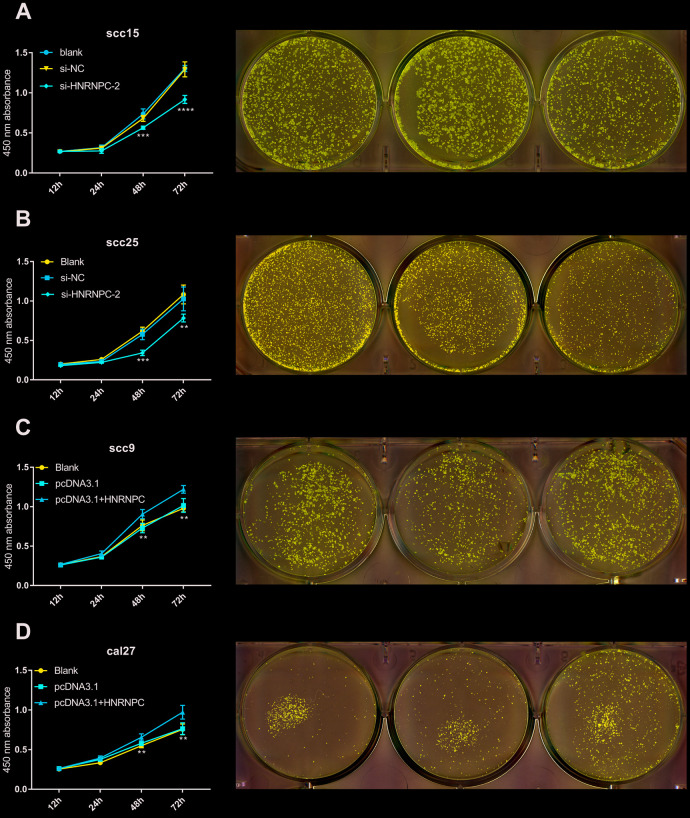
**HNRNPC promoted proliferation in OSCC cell lines.** (**A**) Si-HNRNPC inhibit cell proliferation in scc15 cell line (48h p=0.0002, 72h p<0.0001). (**B**) Si-HNRNPC inhibit cell proliferation in scc25 cell line (48h p=0.0001, 72h p=0.0073). (**C**) Overexpression of HNRNPC promoted proliferation in scc9 cell line (48h p=0.0021, 72h p=0.0035). (**D**) Overexpression of HNRNPC promoted proliferation in cal27 cell line (48h p= 0.0018, 72h p= 0.0022).

**Figure 6:**
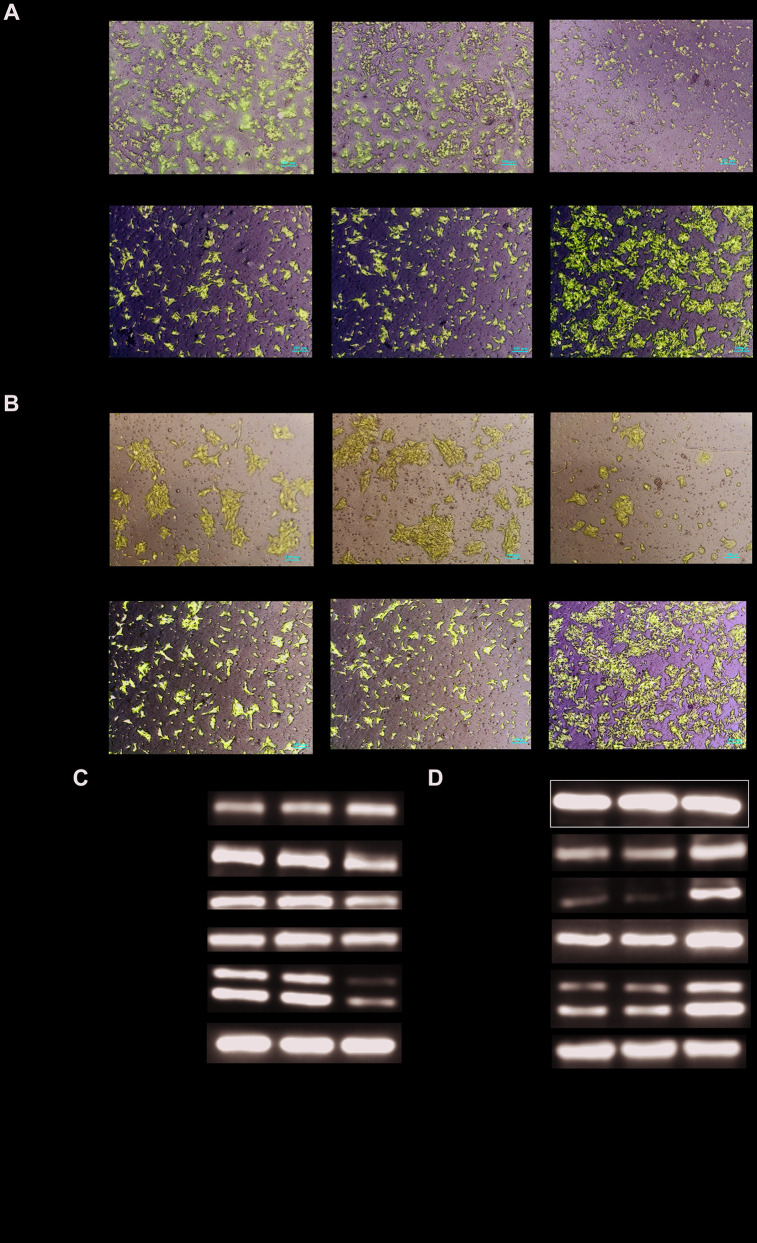
**Detection of migration and invasion abilities.** (**A**, **B**) Migration and invasion abilities were detected in scc15 cell line and cal27 cell line. Overexpression of HNRNPC promoted OSCC cells migration and invasion, and it was oppositely in knockdown of HNRNPC. (**C**, **D**) EMT markers were detected with Western bolt assay. Activation of EMT pathway accelerated the migration and invasion of OSCC.

**Figure 7:**
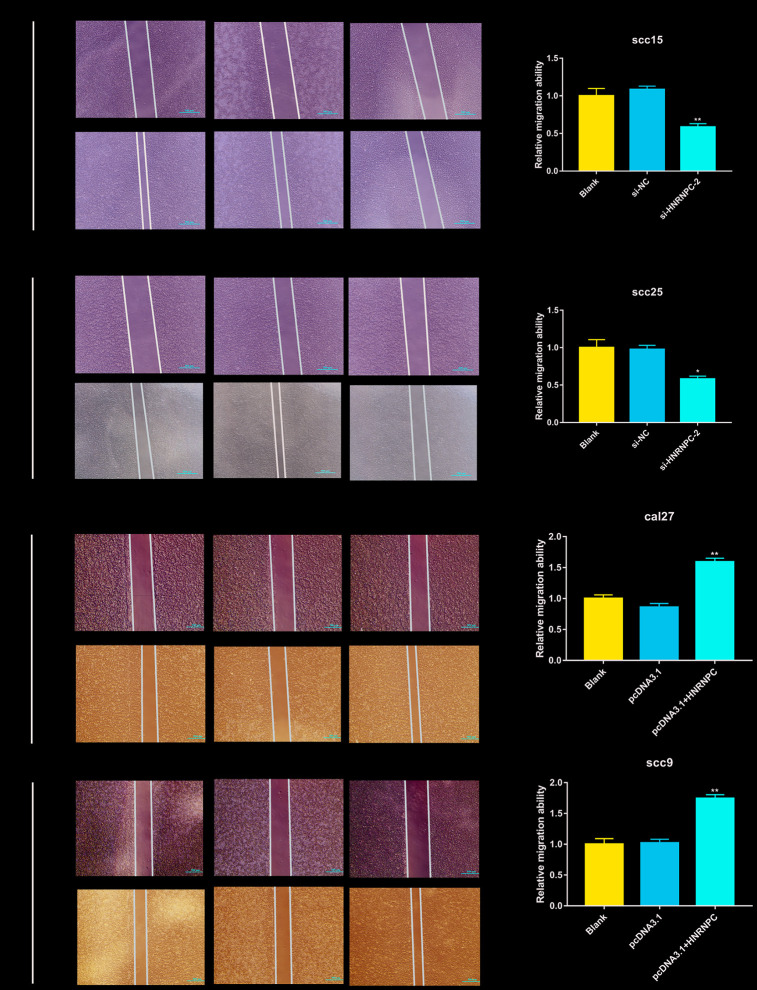
**Scratch wound healing assay in OSCC cell lines.** Scratch wound healing assay were used to evaluate the migration of scc15 cell line (**A**, p= 0.0079), scc25 cell line (**B**, p= 0.0136), cal27 cell line (**C**, p= 0.0068) and scc9 cell line (**D**, p= 0.0066).

To determine the tumorigenic role of HNRNPC in OSCC, the HNRNPC was silenced with siRNA in scc15 and scc25 cells, and overexpression with pcDNA3.1(pcDNA3.1+ HNRNPC) in scc9 and cal27 cells. And the transfection efficiency of HNRNPC was detected with Western blot assay (Supplementary Figure 3A, 3B). The growth of cells was detected via CCK-8 and colony formation assays. As shown in the results, knockdown of HNRNPC significantly delayed cell proliferation and reduced the clonogenicity in scc15 and scc25 cells (Figure 5A, 5B). Oppositely, upregulation of HNRNPC promoted cell proliferation (Figure 5C, 5D). Furthermore, Transwell assay and scratch wound healing were performed to detect cell migration and invasion ability. Migration and invasion ability of OSCC cells were significantly promoted by the overexpression of HNRNPC and inhibited by the knockdown of HNRNPC in scc15 and cal27 cells (Figure 6A, 6B). The results of migration and invasion ability were similar in scc9 and scc25 cell lines (Supplementary Figure 4A, 4B). Brabletz et al. demonstrated that EMT process played a significant tumorigenic role in cancers [17]. Consequently, EMT process pathway-related markers were detected by western blot (Figure 6C, 6D, Supplementary Figure 4C, 4D). Results showed that overexpression of HNRNPC triggered EMT via enhancement of N-cadherin, MMP9, Vimentin and inhibition of E-cadherin in scc9 and cal27 cells. And it is oppositely in knockdown HNRNPC cells. Furthermore, the results of scratch wound healing assay were consistent with Transwell assay (Figure 7). Statistical analysis was performed in SSPS software (Supplementary Figure 5).

## DISCUSSION

OSCC, as the most common oral cancer, poses a great challenge to the medical profession because of its high recurrence rate and low 5-year overall survival rate [18]. Recent researches indicate that m6A is a vital regulator in the initiation and progression of human cancer [19, 20]. However, there are fewer studies to investigate the tumorigenic role of m6A in OSCC. Therefore, it will be helpful to understand the initiation and progression of OSCC to explore the role of m6A.

In our study, 13 m6A-related genes were extracted from TCGA, and m6A levels in RNA were upregulated in tumor tissues compared with normal adjacent tissues, which showed that abnormal m6A modification was closely related to tumorigenesis in OSCC. Furthermore, cluster analysis combined with PCA were performed according to m6A-related genes expression. The results revealed that m6A-related genes expression level can distinguish OSCC patients. Subsequently, a cox regression model was constructed in our study, and the risk score showed no significantly association with clinical parameters except differentiation grade. However, similar study in head and neck squamous cell carcinoma (HNSCC) indicated that a cox model involved in HNRNPC and YTHDC2 was associated with age, gender, stage and grade [21]. This may be due to differences in tumor composition ratio and sample size. In our study, the cox formula included HNRNPC, METTL14, YTHDF2 and ALKBH5, indicating these genes might be related with prognosis of OSCC patients and play an oncogenic role in OSCC. Interestingly, univariate and multivariate cox regression analysis suggested that only HNRNPC was related to prognosis in OSCC. In addition, higher HNRNPC expression levels were positively correlated with advanced clinical stage and lymph node metastasis and meant undesirable overall. Subsequently, our study indicated that HNRNPC promoted proliferation, migration and invasion in OSCC in vitro. These findings totally suggested that HNRNPC might play a tumorigenic role in OSCC. In addition, HNRNPC also promoted cell proliferation and inhibited apoptosis by bounding to primary miR-21 (pri-miR-21) directly and promoted miR-21 expression in glioblastoma [22]. Increasing number of studies demonstrated that EMT was markedly relevant to OSCC progression [23–25]. In our study, expression of N-cadherin, MMP9, Vimentin were up-regulated and E-cadherin was down-regulated after overexpression of HNRNPC. It had the opposite effect after knockdown of HNRNPC. N-cadherin, MMP9, Vimentin and E-cadher are significant markers of EMT. These results showed that HNRNPC might promoted OSCC progression via EMT. In addition, other enrolled in cox formula m6A-related genes METTL14, YTHDF2, ALKBH5 played an oncogenic role in various cancers in a m6A- dependent manner [19, 26, 27]. Unfortunately, there are fewer studies reported that they are related to OSCC. The roles of these genes in OSCC needs further study.

In our present study, a risk cox regression was established on basis of m6A-related genes expression and 4 molecules were enrolled in risk model which is significantly relevant to overall survival rate in OSCC. In addition, risk score may be an independent prognostic biomarker according to univariate and multivariate cox regression analysis. Finally, univariate, multivariate cox regression analysis and survival analysis indicated that HNRNPC may play a tumorigenic role in OSCC. Consequently, the expression levels and functions of HNRNPC were verified in OSCC cells which showed that HNRNPC promoted cell proliferation, migration, and invasion in vitro.

Though the study might have crucial clinical importance, we still need to consider several limitations. First, in terms of sample numbers, both the TCGA database and clinical specimens which were collected at Nanfang Hospital are far from inadequate. Therefore, more information need be harvested to verify its accuracy and further studied in vivo and in vitro should be performed to investigate the function and mechanism of several m6A biomarkers in OSCC.

## MATERIALS AND METHODS

### Data preparation

The transcriptome profiling primitive datum of OSCC including oral cavity, floor of mouth, palate, buccal mucosa, the anterior 2/3 of the tongue, gingiva and so on were downloaded from The Cancer Genome Atlas(TCGA) database (https://portal.gdc.cancer.gov/) through GDC Data Transfer Tool. Total of 319 OSCC samples and 32 controls were downloaded. 2 samples were excluded because of its half-baked clinical data. Eventual, 317 OSCC samples and 32 normal controls were enrolled in our study.

### Patients, sample collection

80 pairs oral squamous cell carcinoma specimens and normal adjacent tissues were collected at Nanfang Hospital, Southern Medical University (Guangzhou, China), and written informed consent was obtained from all patients. The anatomical locations of oral squamous cell carcinoma included buccal, tongue, base of mouth, gingiva. Normal adjacent tissues were located at least 1.5 cm from the edge of the tumor. All tumor and normal adjacent tissues were respectively confirmed as squamous cell carcinoma and normal tissues pathologically. All OSCC samples were divided into high expression and low expression group according to median value of HNRNPC mRNA expression levels. Subsequently, correlation analysis between HNRNPC expression levels and clinical parameters was explored.

### M6A-related genes obtaining and differential analysis

All m6A-related genes including METTL3, METTL14, WTAP, KIAA1429, RBM15, ZC3H13, YTHDC1, YTHDC2, YTHDF1, YTHDF2, HNRNPC, FTO, ALKBH5 were extracted from transcriptome profiling in R software (Version 3.6.1). Meanwhile, differentially expressed gene analysis was performed in R software. Differences were considered significant at p <0.05 *; p <0.01**; p <0.001***. The heatmap and volcano were constructed by the ggplots package in R software.

### Cluster analysis and principal component analysis

Cluster analysis was performed according to m6A-related genes expression profile, and the results of cluster analysis were used to make a principal component analysis (PCA). Furthermore, clinical data and survival time were extracted from 317 OSCC samples. Then, the correlation analysis between clinical traits and clustering results were carry out in R software. Finally, the heatmap and survival chart were constructed by the ggplots package in R software.

### Cox risk regression establishment

The m6A-related genes primitive data were transformed and normalized in a log2(x+1) manner. Prognosis associated factors were selected by univariate cox regression. Subsequently, we performed cox regression analysis combined with LASSO regression to establish a risk model and the penalty regularization parameter lambda (λ) was chosen through the cross-validation routine with an n-fold equal to 10 by using R package glmnet [28]. Meanwhile, *Lambda.min* was identified to pick out the variables. Finally, 4 m6A-related genes were enrolled in risk cox regression and survival analysis, scatter diagram and heatmap were performed in R software according to the risk score for each patient. Moreover, univariate and multivariate cox regression were performed to analyze whether the risk score was an independent prognostic factor.

### Cell culture

The human OSCC cell lines scc9, scc15, scc25, cal27 and the normal oral epithelial cell line HOK were obtained from the Institute of Antibody Engineering, Southern Medical University (Guangzhou, China). HOK was cultured in MEM (Gibco, Cat# C12571500BT-10), scc9 in Dulbecco’s modified Eagle’s medium F12(DMEM/F12) (Gibco,Cat#C11330500BT), scc15, scc25 in DMEM(Gibco,Cat#11995500TB) and cal27 in α-MEM (Gibco,Cat# C12571500BT-10). All cell lines were supplemented with 10% fetal bovine serum (FBS, PAN-Biotech,Cat#ST30-3302) at 37 °C with 5% CO_2_.

### RNA extraction and RT-qPCR

Tissue blocks were collected from NanFang Hospital and saved in RNA WAIT (Solarbio, Cat# SR0020) at -80°C. It was broken up in ultrasonic instruments and total RNA were extracted from tissues and cells following the TRIzol (Takara, Cat# 9109) manufacture’s instruction. The same amount of total RNA was reverse to cDNA according to the Reverse Transcription Kit manufacturer’s protocol (Vazyme, Cat# R212-02). The abundance of interested genes in OSCC samples was quantified by RT-qPCR. For each gene, expression levels were normalized to GAPDH. Experiments were performed in triplicate and results displayed as mean values ± S.E. Details of primer sequences were listed as follow: HNRNPC Forward primer (5’-3’): GCCAGCAACGTTAC CAACAA; Reverse primer (5’-3’): TGAACAGAGCA GCCCACAAT. GAPDH Forward primer (5’-3’): CGCTGAGTACGTCGTGGAGTC; Reverse primer: (5’-3’) GCTGATGATCTTGAGGCTGTTGTC.

### M6A RNA methylation quantification

Overall methylation m6A content was measured by using m6A RNA Methylation Quantification Kit (Epigentek, Cat# P-9005-48) according to manufacturer’s instruction. Briefly, 200ng total RNA was inputted in per reaction following the detection antibody solution was added into per reaction respectively. The m6A level was quantified by colorimetry, and the absorbance of each reaction was measured at 450 nm.

### Immunohistochemistry

OSCC and adjacent normal tissues samples were fixed with 4% formaldehyde, dehydration as well as wax immersion, embedded in paraffin and finally cut into 4 μm sections. The tissue sections were dewaxing and rehydration in xylene and graded ethanol. Then sections were treated for 10min with 3% hydrogen peroxide for 10min to block endogenous peroxidase. Subsequently, 0.01 M citrate buffer (pH 6.0) was performed to antigen retrieval for 15min in pressure cooker. Furthermore, the sections were incubated with the primarily antibody at 4°C overnight and secondary antibody 1h at room temperature. Finally, the sections were visualized with 3,3’-diaminobenzidine (DAB). 3 independent pathologists with no prior knowledge of patient evaluated immunohistochemical staining. Extent of staining was scored on a scale from 0 to 4, corresponding to the percentage of immune-reactive tumor cells (0%, 1–5%, 6–25%, 26–75%, and 76–100%, respectively). Staining intensity was scored as negative (score = 0), weak (score= 1), or strong (score = 2). A score ranging from 0 to 8 was calculated by multiplying the score for staining extent with that for intensity. Final grades (negative, 1+, 2+, and 3+) were assigned to each specimen with scores of 0–1, 2–3, 4–5, and 6–8, respectively [29]. When the quantitative scores did not match among the 3 pathologists, the average score of 3 pathologists would be used to evaluated immunohistochemical staining.

### Cell transfection

All the small interfering RNAs (siRNAs, TINGKE, 100mM) and pcDNA3.1 expression for HNRNPC were designed and synthesized. SiRNAs and pcDNA3.1 expression HNRNPC(pcDNA3.1+HNRNPC, TINGKE, 100mM) were thrown into cells followed the protocol of lipofectamine 3000 (Invitrogen, Cat# L3000-015). Cell transfection was performed in 6-well, and each well added 2500ng siRNA or HNRNPC overexpression vector. RNA and protein were collected after 2-4 days. And the siRNA sequence wa listed as follow:si-HNRNPC-1; sense 5’-3’: CAACGGGACUAUUAUGAUA, antisense 5’-3’: UAUCAUAAUAGUCCCGUUG; si-HNRNPC-2; sense 5’-3’: GCGCUUGUCUAAGAUCAAAUU, antisense 5’-3’: AAUUUGAUCUUAGACAAGCGC.

### Cell viability assay

About 3*10^3^ OSCC cells were cultured in 96-well plates after transfection, cell viability was examined using Cell Counting Kit (Vazyme,Cat#A311-02-AA) at 12, 24, 48 and 72 hours with Biotek synergy HTX. Add 100ul serum-free medium and 10ul cck8 to each well, and then incubating 2h at 37 °C with 5% CO_2_. Finally, absorbance was detected at 450nm.

### Clonogenic assay

The cells were digested with 0.25% trypsase (Solarbio, Cat# T1300) at about 70%-90% confluent and suspended in α-MEM, DMEM or DMEM/F12 with 10% FBS. Then, transfected cells were seeded in 6-well plates at the starting density of 2*10^3^ cells per well at 37°C with 5%CO_2_ for 7-14 days. Furthermore, cells were washed with PBS for 3 times and fixed in 4% paraformaldehyde(Panera, Cat# AAPR12-500) for 15 min, following stained with 0.1% crystal violet staining solution for 20 min. Finally, colony images were taken under an inverted phase-contrast microscope.

### Transwell assay

The matrigel 1:8 dilution was coated at the bottom of the chamber, and put it at 37 °C for 2 hours. 5*10^4^ transfected cells were suspended in 200ul serum-free medium per well, and seed in the upper chambers. 600 μL DMEM or DMEM/F12 with 10% FBS was placed to the bottom wells. In addition, the migration assay was without matrigel in chamber. Furthermore, transfected cells were cultured at 37 °C with 5% CO_2_ for 24-72 h. Subsequently, the cells in the upper side were removed with a cotton swab, and the migration and invasion cells were fixed by 4% formaldehyde and then stained with crystal violet. Finally, inverted microscope was used to count the number of migration and invasion cells.

### Wound-healing assay

The transfected cells were cultured in 6-well plates at a density of 5*10^5 cells/well. When cells grown until reaching a confluence of 90%, a linear wound was generated across the cell monolayer by a sterile 200 μL pipette tip. Furthermore, the cells were washed by PBS 3 times to remove floating cells or debris, and then cultured in serum-free medium with 5% CO_2_ at 37 °C for additional 12-24 hours. Images were taken at 0, 12, 24 hours under an inverted phase-contrast microscope.

### Western blot analysis

OSCC cell lines and tissues protein samples were lysed in RIPA lysis buffer (SIGMA, Cat# R0278). Furthermore, proteins were separated by SDS PAGE gels, transferred to PVDF membranes (Pall, Cat# BSP0161) and then sealed with 5 % skim milk. The primary antibodies were incubated at 4 °C for overnight. Subsequently, goat anti-mouse (Proteintech, Cat# SA00001-1,1:10000) and goat anti-rabbit (Proteintech, Cat# SA00001,1:10000) secondary antibodies for 1 h at room temperature, and finally proteins were quantified by ECL (YEASEN, Cat# 36208ES76) Prime Western Blotting Detection reagent. The information of primary antibodies was as follows. HNRNPC (Abclonal, Cat# A0057,1:1000); E-cadherin (Proteintech, Cat# 20648-1-AP,1:5000); N-cadherin (Proteintech, Cat# 13769-1-AP,1:5000); MMP9 (Proteintech, Cat# 10375-2-AP,1:5000); GAPDH (Proteintech, Cat# 66031-1-1g,1:5000); α-tublin (Proteintech, Cat# 66031-1-1g,1:5000).

### Statistical analysis

All analyses were performed with the SPSS23.0 software (IBM) for statistical analysis. Statistical significance was determined by Student’s t-test. Long rank test was used to analyze survival between two groups. P-values < 0.05 were considered significant. Significant differences were considered at p <0.05 *; p <0.01 **; p <0.001***; p<0.0001****.

### Ethics statement

The study protocol was approved by the Ethics Committees of Nanfang Hospital of Guangdong Province (NO: NFEC-2018-027).

## CONCLUSIONS

In general, our study comprehensively analyzed m6A-related genes and provided a potential therapeutic targets for OSCC. Furthermore, the risk score model constructed in our study may be helpful for predicting the overall survival in OSCC. At last, our functional studies show that HNRNPC may serve as a novel biomarker for tumorigenesis and prognosis of OSCC.

## Supplementary Material

Supplementary Figures
