# Supplementary material for: M6A-related bioinformatics analysis reveals that HNRNPC facilitates progression of OSCC via EMT
Source: Aging (Albany NY). 2020 Jun 11;12(12):11667–84. doi: 10.18632/aging.103333 (PMC7343469; doi:10.18632/aging.103333)
Supplement: Supplementary Figures [file aging-12-103333-s001..pdf]

SUPPLEMENTARY FIGURES

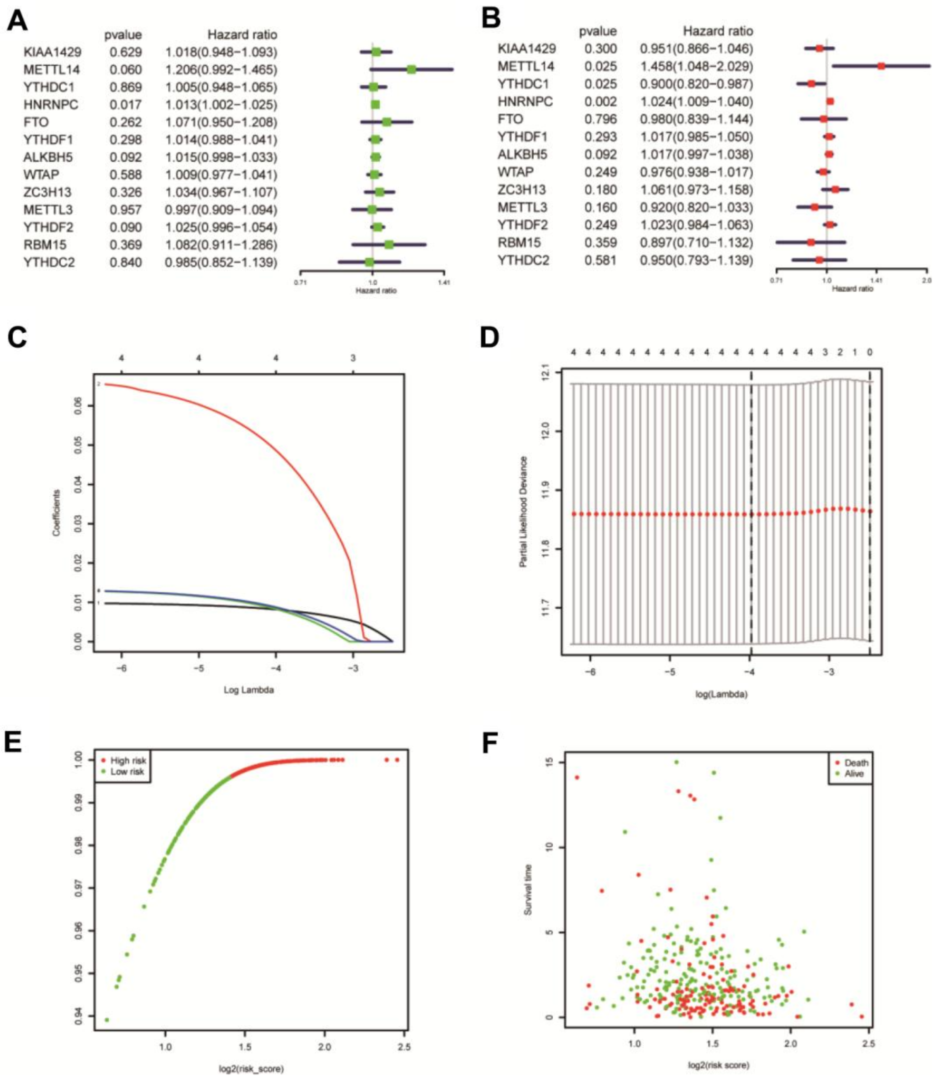

Supplementary Figure 1. LASSO regression and visualization of risk score. (**A**, **B**) Univariate and multivariate cox regression based on 13 m6A related genes. (**C**) LASSO coefficient profiles of the m6A related genes associated with the overall survival of OSCC. (**D**) Partial likelihood deviance was plotted vs. log(c). The vertical dotted line indicated the lambda value with the minimum error and the largest lambda value, where the deviance is within one SE of the minimum. (**E**) Visualization of risk models. The y axis represents percentage and x axis log2(risk score). (**F**) The scatter diagram according to survival time and log2(risk score). The red means death and green live.

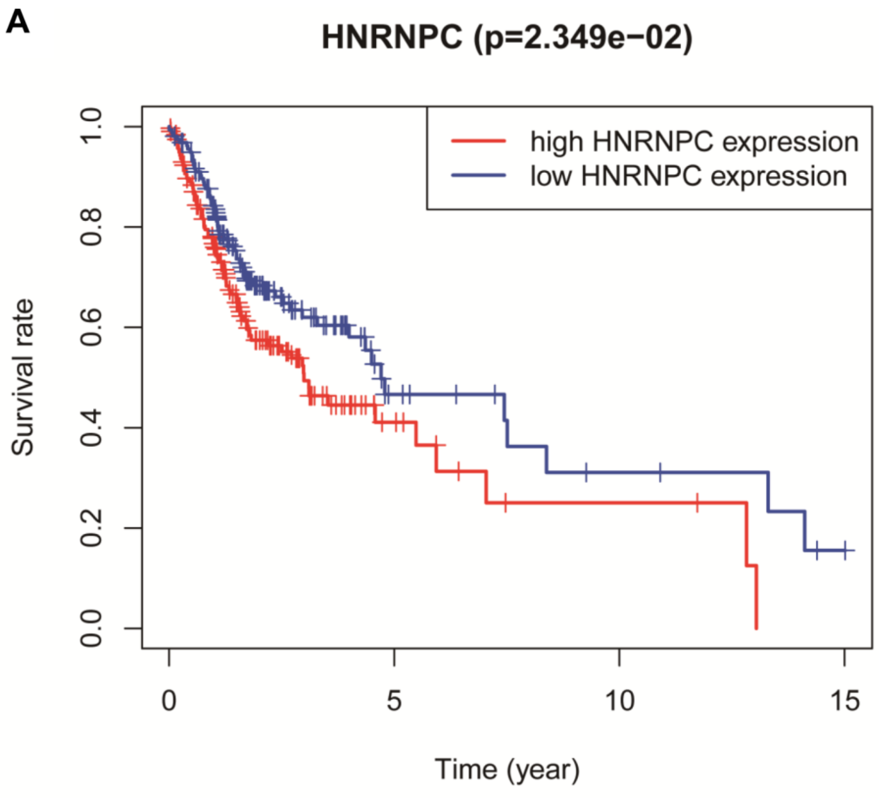

Supplementary Figure 2. HNRNPC survival analysis in TCGA. (**A**) Survival analysis of m6A reader HNRNPC in TCGA (p=0.0234).

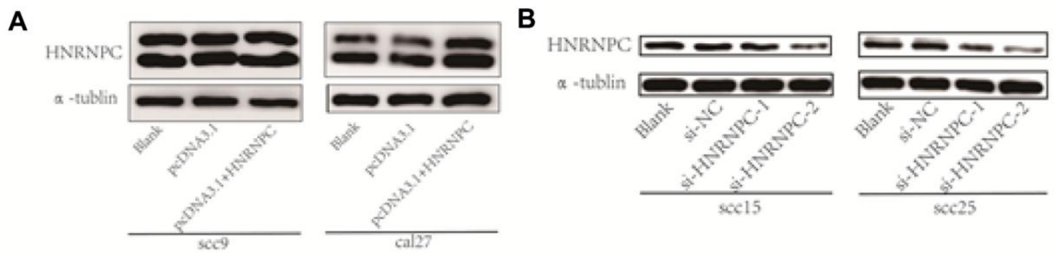

Supplementary Figure 3. Transfection efficiency of HNRNPC. (**A**) Overexpression vector of HNRNPC was transfected in scc9 and cal27 cell lines. (**B**) Si-HNRNPC was transfected in scc15 and scc25 cell lines.

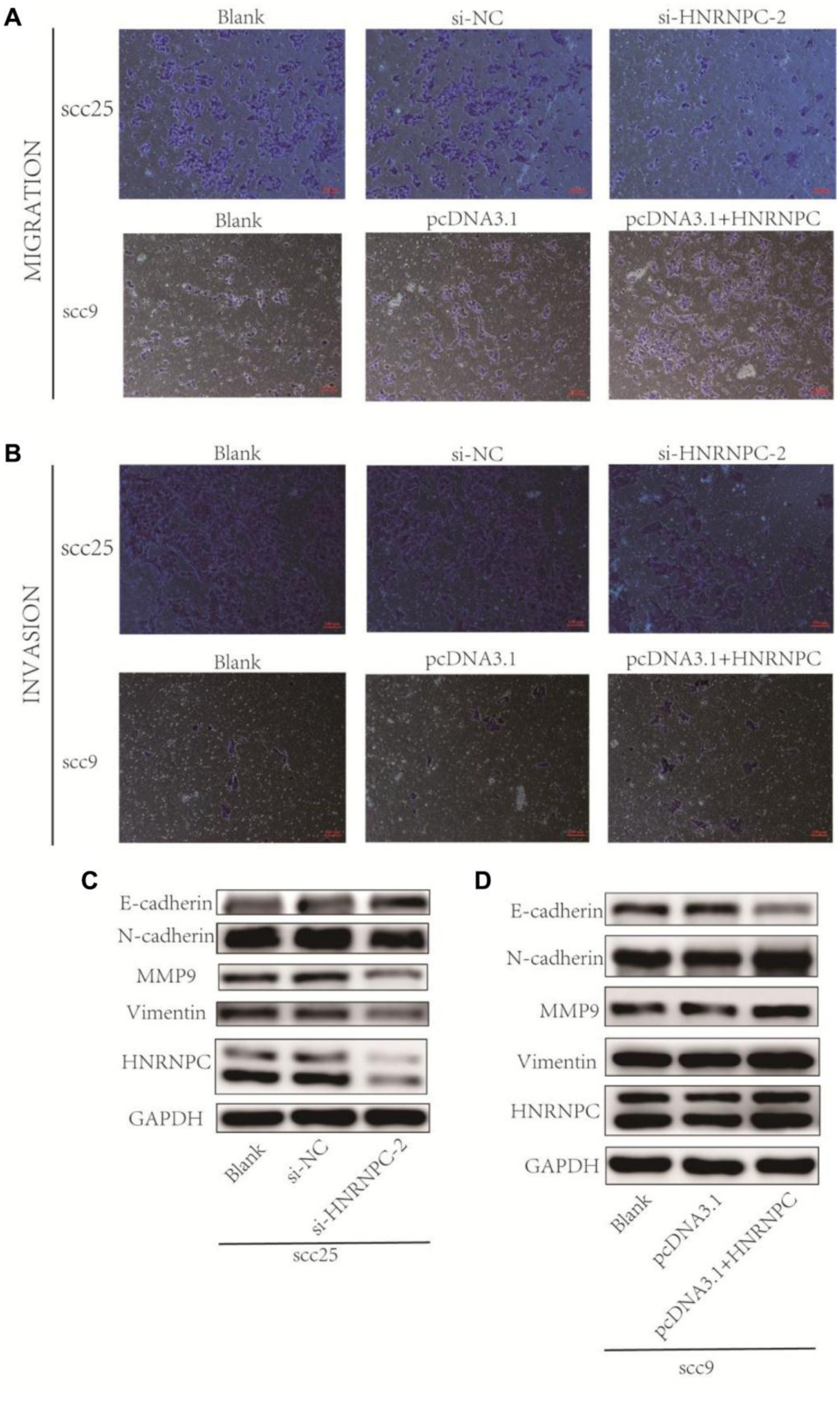

Supplementary Figure 4. Detection of migration and invasion abilities. (**A**, **B**) Migration and invasion abilities were detected in scc25 cell line and scc9 cell line. (**C**, **D**) EMT markers were detected with Western bolt assay.

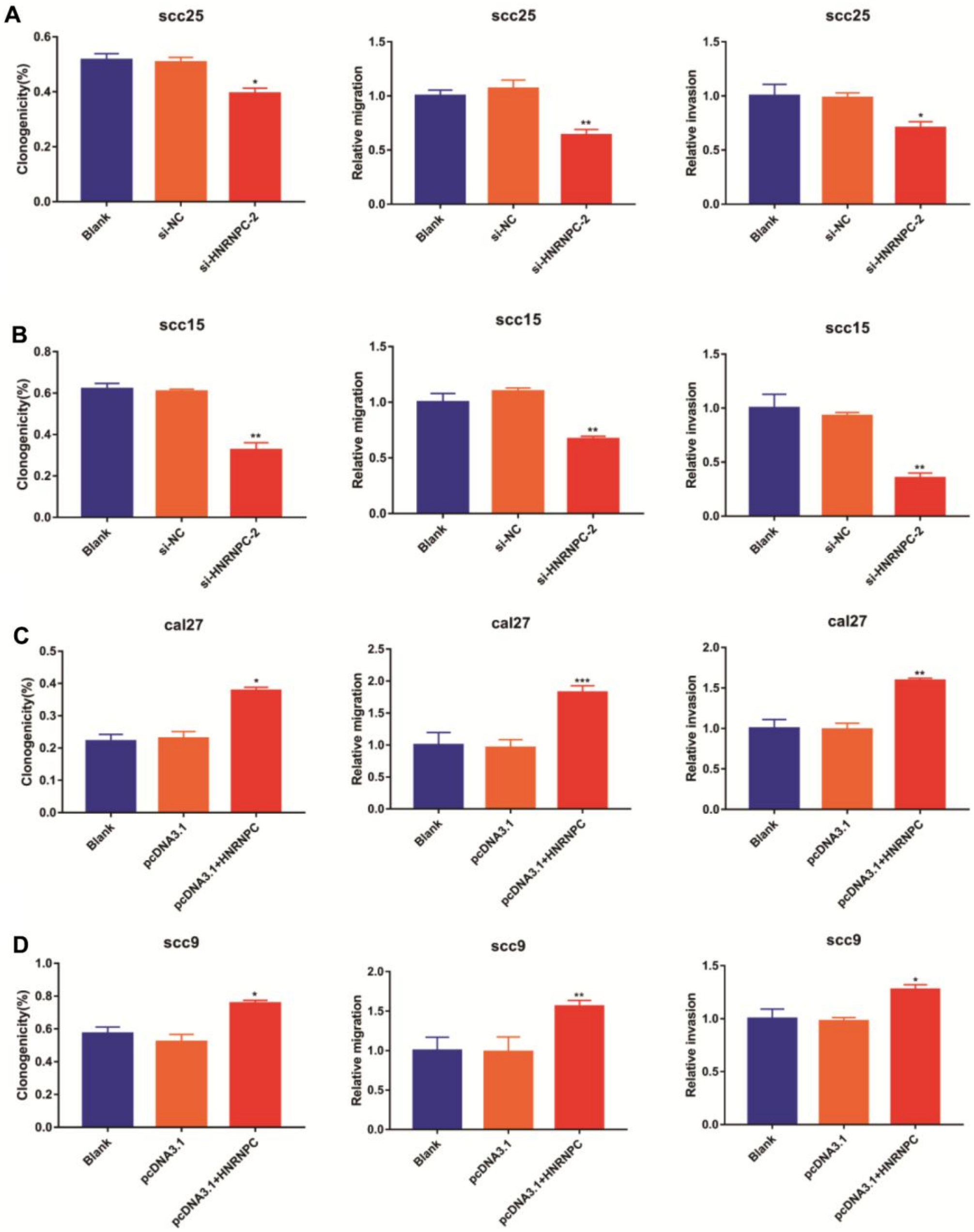

Supplementary Figure 5. Statistical analysis of Clonogenic assay and Transwell assay. (**A**) Clonogenic assay (p=0.0257), migration (p=0.0014) and invasion assay (p=0.0330) in scc25 cell line. (**B**) Clonogenic assay (p=0.0085), migration (p=0.0041) and invasion assay (p=0.0044) in scc15 cell line. (**C**) Clonogenic assay (p=0.0127), migration (p=0.0008) and invasion assay (p=0.0093) in cal27 cell line. (**D**) Clonogenic assay (p=0.0209), migration (p=0.0078) and invasion assay (p=0.0180) in scc9 cell line.
